# Cardiac Sympathetic Modulation in Response to Apneas/Hypopneas through Heart Rate Variability Analysis

**DOI:** 10.1371/journal.pone.0086434

**Published:** 2014-01-22

**Authors:** Florian Chouchou, Vincent Pichot, Jean-Claude Barthélémy, Hélène Bastuji, Frédéric Roche

**Affiliations:** 1 Service de Physiologie Clinique, Pole NOL, CHU Nord, Saint-Etienne, France; 2 EA 4607 SNA-EPIS, Université Jean Monnet, PRES de Lyon, Saint-Etienne, France; 3 Unité NeuroPain, Centre de recherche en Neurosciences de Lyon, INSERM U1028 CNRS UMR5292, Lyon, France; University of Milan, Italy

## Abstract

Autonomic dysfunction is recognized to contribute to cardiovascular consequences in obstructive sleep apnea/hypopnea syndrome (OSAHS) patients who present predominant cardiovascular sympathetic activity that persists during wakefulness. Here, we examined 1) the factors that influence sympathetic cardiac modulation in response to apneas/hypopneas; and 2) the influence of autonomic activity during apneas/hypopneas on CA. Sixteen OSAHS patients underwent in-hospital polysomnography. RR interval (RR) and RR spectral analysis using wavelet transform were used to study parasympathetic (high frequency power: HF^WV^) and sympathetic (low frequency power: LF^WV^ and LF^WV^/HF^WV^ ratio) activity before and after apnea/hypopnea termination. Autonomic cardiac modulations were compared according to sleep stage, apnea/hypopnea type and duration, arterial oxygen saturation, and presence of CA. At apnea/hypopnea termination, RR decreased (p<0.001) while LF^WV^ (p = 0.001) and LF^WV^/HF^WV^ ratio (p = 0.001) increased. Only RR and LF^WV^/HF^WV^ ratio changes were higher when apneas/hypopneas produced CA (p = 0.030 and p = 0.035, respectively) or deep hypoxia (p = 0.023 and p = 0.046, respectively). Multivariate statistical analysis showed that elevated LF^WV^ (p = 0.006) and LF^WV^/HF^WV^ ratio (p = 0.029) during apneas/hypopneas were independently related to higher CA occurrence. Both the arousal and hypoxia processes may contribute to sympathetic cardiovascular overactivity by recurrent cardiac sympathetic modulation in response to apneas/hypopneas. Sympathetic overactivity also may play an important role in the acute central response to apneas/hypopneas, and in the sleep fragmentation.

## Introduction

Epidemiological studies suggest that obstructive sleep apnea/hypopnea syndrome (OSAHS) is common in the general population [1], and provide strong evidence that OSAHS is associated with significantly high cardiovascular morbidity and mortality [2]. Autonomic dysfunction is now recognized to contribute to these cardiovascular consequences [3] in OSAHS patients who present decrease in heart rate variability (HRV) [4] and predominant cardiovascular sympathetic activity that persists during wakefulness [5]. Moreover, sympathetic sleep fragmentation was associated with elevated nocturnal and diurnal systolic blood pressure and higher risk of systolic hypertension [6]. Studying the mechanisms that control sympathetic cardiac modulation in response to apneas/hypopneas by HRV should improve our understanding of the cardiovascular risk factor in OSAHS populations.

OSAHS is characterized by repeated episodes of total (apneas) or partial (hypopneas) upper airway occlusion during sleep, resulting in exaggerated negative intrathoracic pressure and often oxygen desaturation and carbon dioxide retention [3]. At termination, apneas/hypopneas frequently trigger cortical arousals (CA) [7], a process thought to restore pharyngeal dilator muscle tone and airflow. Apneas/hypopneas also elicit oscillations in both parasympathetic and sympathetic cardiac activities that affect RR intervals (RR), characterized by increased parasympathetic activity during apneas/hypopneas and increased sympathetic activity at apnea/hypopnea termination [8]–[10].

The factors that modulate sympathetic cardiac modulation in response to apneas/hypopneas remain unclear, and the research findings are contradictory [8]–[10]. Sympathetic cardiac change in response to apneas/hypopneas during paradoxical sleep has been reported as higher [10] or lower [8], [9] than in other sleep stages, whereas in response to external [11] or internal [12] stimulations, this sympathetic cardiac modulation did not appear to differ according to sleep stage. Although this sympathetic cardiac change was elevated when CA was generated by external [11] or internal [12] stimulations, a relationship between CA and sympathetic cardiac modulation in response to apneas/hypopneas was reported [8], [9] or not [10]. Only one study specifically examined the influence of the type of respiratory events and found no significant effect [8], and another that of hypoxia, and showed a correlation between concomitant minimal oxygen saturation (min SaO_2_) and sympathetic cardiac modulation [10]. To our knowledge, the effect of the duration of respiratory events on sympathetic modulation has never been investigated.

Sympathetic cardiac activity during sleep was also proposed as a potential contributor to CA occurrence following the observation in animals [13], [14] and humans [15], [16] that baroreflex loop stimulation during sleep, an autonomic sensitivity component, could induce CA. During apneas/hypopneas, although RR increase and parasympathetic activity dominates, Bonsignore [17] reported two typical patterns: RR continued to increase during apneas/hypopneas, and RR decreased before apnea/hypopnea termination. However, to our knowledge, no study has examined the relationship between autonomic cardiac activity during apneas/hypopneas and subsequent CA while elevated cardiac sympathetic precede spontaneous CA occurrence [18].

Based on these findings, we first assessed autonomic cardiac modulation at obstructive apnea/hypopnea termination during all-night sleep and its relationship to electroencephalographic (EEG) cortical reactivity, sleep stage, min SaO_2_, and apnea/hypopnea duration and type (hypopneas vs. apneas). We hypothesized that 1) sympathetic cardiac activation should be more intense when apneas/hypopneas give rise to CA or 2) when it is accompanied by a deeper hypoxia, but 3) with no difference between sleep stages. We also hypothesized 4) a rise in cardiac sympathetic modulation with longer apnea/hypopnea duration, and 5) with apnea versus hypopnea type. We also assessed the relationship between autonomic activity level during apneas/hypopneas and subsequent CA occurrence. We hypothesized that 6) high sympathetic cardiac activity during apneas/hypopneas should be associated with facilitated CA occurrence.

## Methods

### Subjects

Sixteen untreated OSAHS patients (3 women, Table 1) were studied (Clinical Trial registration: NCT01135303). Exclusion criteria were any known cardiac abnormalities or neurological disease and being under adrenergic receptor blockers as previously published [8], [10]. Seven hypertensive patients were included, since they were not under adrenergic receptor blockers but diuretic and angiotensin-converting enzyme inhibitors. No other cardiac or respiratory disorder was present (Table 1). The study was approved by the Local Ethics Committee (CCP Saint-Etienne Sud) and performed under informed consent according to the Declaration of Helsinki. All participants provided their written informed consent to participate in this study.

**Table 1:** Demographic and polysomnographic parameters in the study group (mean ± SEM).

	Mean	SEM
Age (yrs)	48.8	2.9
BMI (kg/m^2^)	31.7	3.2
Total sleep time (min)	403.0	6.5
Wake after sleep onset (min)	26.0	2.2
Sleep efficiency (%)	90.7	1.34
S1 (%)	2.6	0.4
S2 (%)	53.2	2.3
SWS (%)	16.7	1.5
PS (%)	20.1	2.3
Cortical arousal index (n/hr)	49.8	14.8
Obstructive apnea/hypopnea index (n/hr)	35.5	3.8
Obstructive apnea index (n/hr)	10.9	2.2
Hypopnea index (n/hr)	24.6	3.4
Oxygen desaturation index (n/hr)	27.3	3.5
Minimum oxygen saturation (%)	79.3	1.4

### Polysomnographic recordings

Standard polysomnographic recordings were performed using an Embla Recorder (Embla®, Broomfield, USA) according to AASM recommendations [19]. Three electroencephalogram (EEG) leads (F_3_-F_4_; C_3_-C_4_; O_1_-O_2_), three electromyographam (EMG) channels (chin and both legs), two electro-oculogram (EOG) channels (E_1_-M_2_; E_2_-M_2_), and one EKG channel were used. EEG and EOG data were collected at 100 Hz sampling rate and EKG data were sampled at 200 Hz [20]. Respiration was monitored by nasal cannula (200 Hz), two piezoelectric belts (10 Hz) for chest and abdominal efforts, and one oxygen saturation channel (1 Hz). Electrophysiological and respiratory data were recorded continuously from 11:00 pm to 07:00 am and stored for off-line analysis.

### Sleep analysis

To determine in which sleep stage every apneas/hypopneas occurred during the whole night, sleep stages were visually scored according to AASM 2007 criteria [19]. Hypnograms were scored based on 30-s epochs and differentiated into stage 2 (S2), slow-wave sleep (SWS), and paradoxical (REM) sleep (PS) (Table 1). CA were defined as bursts of wakefulness-related cortical activity lasting more than 3 s. Obstructive apneas were defined as complete cessation of airflow (on airflow cannula) in the presence of respiratory effort lasting ≥10 s. When respiratory effort partially or totally ceased, apneas were scored as mixed apnea or central apnea, respectively, and eliminated. Hypopneas were defined as a ≥50% airflow reduction compared with the 3 preceding breaths. Oxygen desaturations were defined as a ≥3% decrease in arterial oxygen saturation.

### RR interval analysis

Peak-to-peak EKG signals were analyzed to detect R waves in the QRS complex using Matlab (MathWorks, Naticks, MA, USA). EKG data were first visually selected. All undetected QRS, ectopic beats, and artifacts were corrected on EKG data. If correction was not possible for at least one heartbeat on selected periods, data were eliminated (236 excluded for artifacts and ectopic beats, 7.4% of a total of 3191 apneas/hypopneas). RR was then analyzed for each apnea/hypopnea on 32 heartbeats before and after apnea/hypopnea termination. Statistical comparisons were performed on the mean of 3 minimal RR between 2 and 7 heartbeats post- apnea/hypopnea termination (mean RR) compared with the mean of the 10 RR pre- apnea/hypopnea termination.

### Spectral analysis of heart rate

Wavelet analysis was performed on the RR signal to analyze both the time and frequency domain, using the mother function Daubechies 4. The wavelet analysis signal allows quantifying temporal changes in the signal frequency contents. Starting from the initial Daubechies 4 function, a family of functions was built by dilatation and translocation to constitute a wavelet frame. A serial list of wavelet coefficients was used to represent the correlation between the signal and the selected wavelet at different analysis levels (or different frequency ranges) along the entire signal. The first levels (2, 4, 8) correspond to a wavelet analysis conducted with a small dilatation factor applied to 2, 4 and 8 RR, respectively. Thus, these levels represent high-frequency variations in the signal. The last levels (16, 32, 64, etc.) correspond to a wavelet analysis conducted with a large dilatation factor applied to 16, 32, and 64 RR, respectively. Consequently, these levels represent low-frequency variations in the signal.

**Figure 1:**
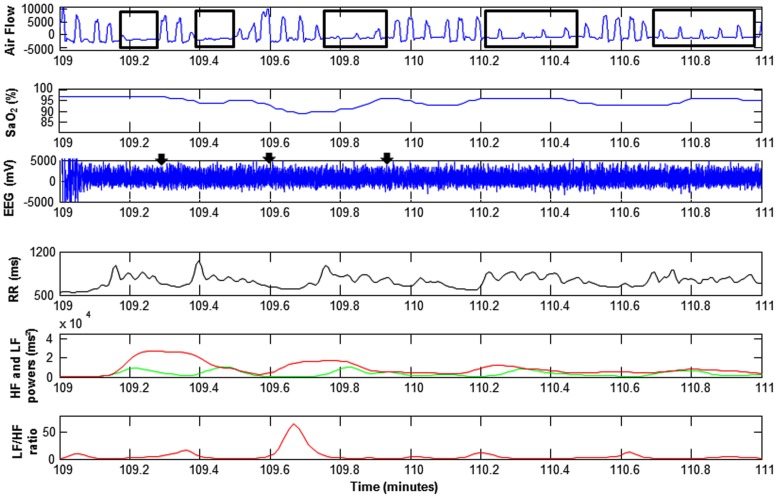
Representative examples of cardiac sympathetic modulation in response to apneas/hypopneas: RR intervals (RR), low (LF: red line) and high frequency (green line: HF) powers and LF/HF ratio (red line). Obstructive hypopnea and apnea were observable on air flow (black rectangles), with oxygen desaturations (SaO_2_≥3%), and sometimes with cortical arousals. Cortical arousals were represented by black arrows, were detected on 3 electroencephalographic leads F_3_-F_4_; C_3_-C_4_; O_1_-O_2_ defined as bursts of wakefulness-related cortical activity lasting more than 3 s scored by expert in sleep studies based on 30-s epochs, but only C_3_-C_4_ lead was represented.

According to the Task Force of the European Society of Cardiology and the North American Society of Pacing and Electrophysiology [21], squared wavelet coefficients at levels 2, 4, and 8 (high frequency: HF^WV^) were used to assess parasympathetic modulation. Wavelet power coefficients at levels 16 and 32 (low frequency: LF^WV^) were used to assess sympathetic modulation. An LF^WV^/HF^WV^ ratio was calculated as the ratio between the wavelet power coefficient at levels 16 and 32 on the one hand and the sum of the wavelet power coefficients at levels 2, 4, and 8 on the other to obtain a marker of autonomic nervous system balance (see Figure 1). This analysis was performed on 32 RR before and after each apnea/hypopnea termination [11].

### Statistical analysis

Data were analyzed using Statview® (SAS Institute, Inc. Cary, NC, USA) software. Mean RR, HF^WV^, LF^WV^, and LF/HF^WV^ ratio values were submitted to different repeated-measures analyses of variance (ANOVA). The first factor was time (pre- vs. post-apnea/hypopnea termination) in ANOVAs and the second factor was, successively: (a) sleep stage (S2 vs. SWS vs. PS) in the first ANOVA; (b) type (hypopneas vs. apneas); (c) and (d) hypopnea and apnea duration, respectively (short: 10 s≤apneas/hypopneas<20 s vs. medium: 20 s≤apneas/hypopneas<30 s vs. long: apneas/hypopneas≥30 s); (e) min SaO_2_ (light: min SaO_2_≥90 %, medium: 90>min SaO_2_>85%, and deep: min SaO_2_≤85%); and (f) CA (arousal vs. no reaction).

To assess whether CA occurrence is independently associated with autonomic activity during apneas/hypopneas, logistic multivariate regressions were performed between each autonomic cardiac index during apneas/hypopneas (expressed in continuous values and tertiles) and the CA presence, adjusted to minimum and mean SaO_2_, type, sleep stage, interval inter-events, and subject. Risks were expressed as odds ratios (OR) with 95% confidence interval (95% CI), differences were considered significant when p<0.05, and values were expressed as mean ± SEM.

## Results

### Number of respiratory events studied

Of a total of 3191 apneas/hypopneas, 2955 were included in the analysis, with 236 excluded for artifacts and ectopic beats. For the whole group, 186±64 events per subject were selected, of which 128±49 were in S2, 19±17 in SWS, and 40±20 in PS. Due to lack of apneas/hypopneas during SWS in some OSAHS patients, statistical analysis was performed on 7 subjects for comparison between sleep stages. Due to lack of apneas/hypopneas with deep hypoxia (SaO_2_≤85%) and long duration (30 s) in some subjects, statistical analysis was performed on 10 subjects to compare min SaO_2_ levels, 11 subjects to compare apnea durations, and 13 subjects to compare hypopnea durations.

### Occurrence of cortical arousal in response to respiratory events

In the 16 OSAHS patients, more than three-quarters of RE (78.7±4.4%) produced CA distributed evenly across sleep stages, with no significant effect of sleep stage on their incidence (n = 7, S2: 79.1±9.0%, SWS: 79.5±179% and PS: 69.6±16.8%; Friedman's X(2) = 0.857; p = 0.651). However, an effect on CA occurrence was noted according to RE type (hypopnea: 76.3±7.7% and apnea: 86.0±5.0%; Wilcoxon rank Z = −5.01; p<0.001). RE duration also modulated CA occurrence in apneas (n = 11, short: 78.8±17.8 %, medium: 83.7±18.9% and long: 98.9±2.4%; Friedman's X(2) = 11.42; p = 0.003) and hypopneas (n = 13, short: 68.8±15.6%, medium: 80.2±10.4% and long: 82.6±11.8%; Friedman's X(2) = 7.17; p = 0.028). However, hypoxia did not appear to influence CA occurrence (n = 10, light: 73.3±7.8%, medium: 77.1±12.5% and deep: 77.1±11.9%; Friedman's X(2) = 2.51; p = 0.285). Thus, the percentage of CA occurrence increased with RE severity and duration, but not according to sleep stage or hypoxia.

### Oxygen desaturation during respiratory events

Mini SaO_2_ did not differ according to the presence or absence of CA (with CA: 88.9±2.8%, without CA: 88.5±3.1%, Wilcoxon rank Z = −0.378; p = 0.705), or according to sleep stage (S2: 88.5±2.6%, SWS: 87.5±4.0%, PS: 87.8±2.3%, Friedman's X(2) = 1.455; p = 0.483), type (hypopneas: 89.5±2.4%, apneas: 87.8±3.4%, Wilcoxon rank Z = −1.394; p = 0.163), or RE duration (hypopneas: short: 90.0±2.5%, medium: 90.0±2.3% and long: 89.5±2.4%; Friedman's X(2) = 0.676; p = 0.708 and apneas: short: 88.8±2.6%, medium: 89.0±3.5% and long: 88.4±3.9%; Friedman's X(2) = 0.092; p = 0.954).

### RR intervals and wavelet analysis at apnea/hypopnea termination

**Figure 2:**
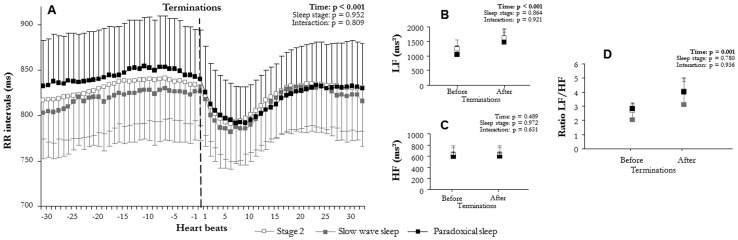
Comparison of autonomic modulation at apnea/hypopnea terminations according to sleep stage: (A) RR intervals (RR), (B) low (LF^WV^) and (C) high frequency (HF^WV^) wavelet power, and (D) LF^WV^/HF^WV^ ratio before and after apnea/hypopnea terminations according to stage 2 sleep, slow wave sleep, and paradoxical sleep (mean ± SEM). An overall effect on RR intervals, LF^WV^, and LF^WV^/HF^WV^ ratio appeared between before and after the apnea/hypopnea terminations. Apneas/hypopneas induced a sympathetic-dependent RR decrease, which was not modulated by sleep stage.

**Figure 3:**
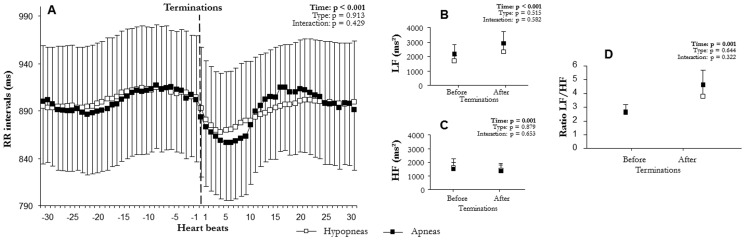
Comparison of autonomic modulation at terminations according to apneas and hypopneas: (A) RR interval (RR), (B) low (LF^WV^) and (C) high frequency (HF^WV^) wavelet power, and (D) LF^WV^/HF^WV^ ratio before and after the apnea/hypopnea terminations according to hypopneas and apneas (mean ± SEM). An overall effect on RR intervals, LF^WV^, and LF^WV^/HF^WV^ ratio appeared between before and after apnea/hypopnea terminations. Apneas/hypopneas induced a sympathetic-dependent RR decrease, which was not modulated by respiratory event type.

**Figure 4:**
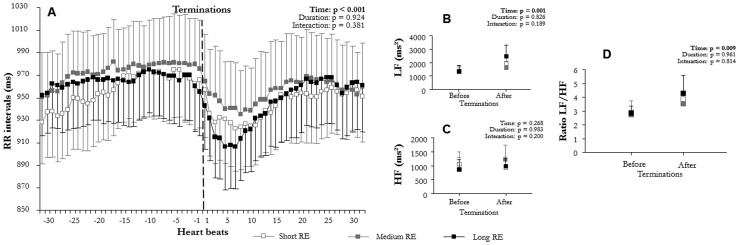
Comparison of autonomic modulation at hypopnea terminations according to hypopnea duration: (A) RR intervals (RR), (B) low (LF^WV^) and (C) high frequency (HF^WV^) wavelet power, and (D) LF^WV^/HF^WV^ ratio before and after terminations according to hypopnea duration: short (10 s≤hypopneas<20 s), medium (20 s≤hypopneas<30 s), and long (hypopneas≥30 s) (mean ± SEM). An overall effect on RR intervals, LF^WV^, and LF^WV^/HF^WV^ ratio appeared between before and after hypopnea terminations. Hypopneas induced a sympathetic-dependent RR decrease, which was not modulated by hypopnea duration.

**Figure 5:**
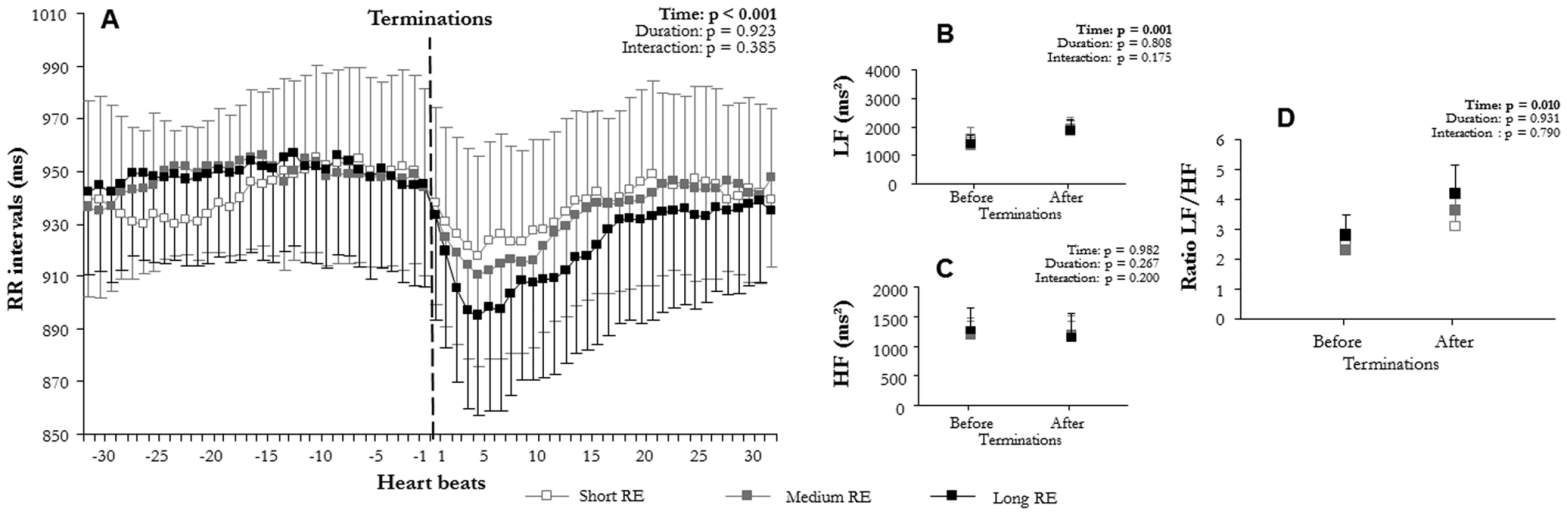
Comparison of autonomic modulation at terminations according to apnea duration: (A) RR intervals (RR), (B) low (LF^WV^) and (C) high frequency (HF^WV^) wavelet power, and (D) LF^WV^/HF^WV^ ratio before and after apnea terminations according to apnea duration: short (10 s≤apneas<20 s), medium (20 s≤apneas<30 s), and long (apneas≥30 s) (mean ± SEM). An overall effect on RR intervals, LF^WV^, and LF^WV^/HF^WV^ ratio appeared between before and after apneas terminations. Apneas induced a sympathetic-dependent RR decrease, which was not modulated by apnea duration.

**Figure 6:**
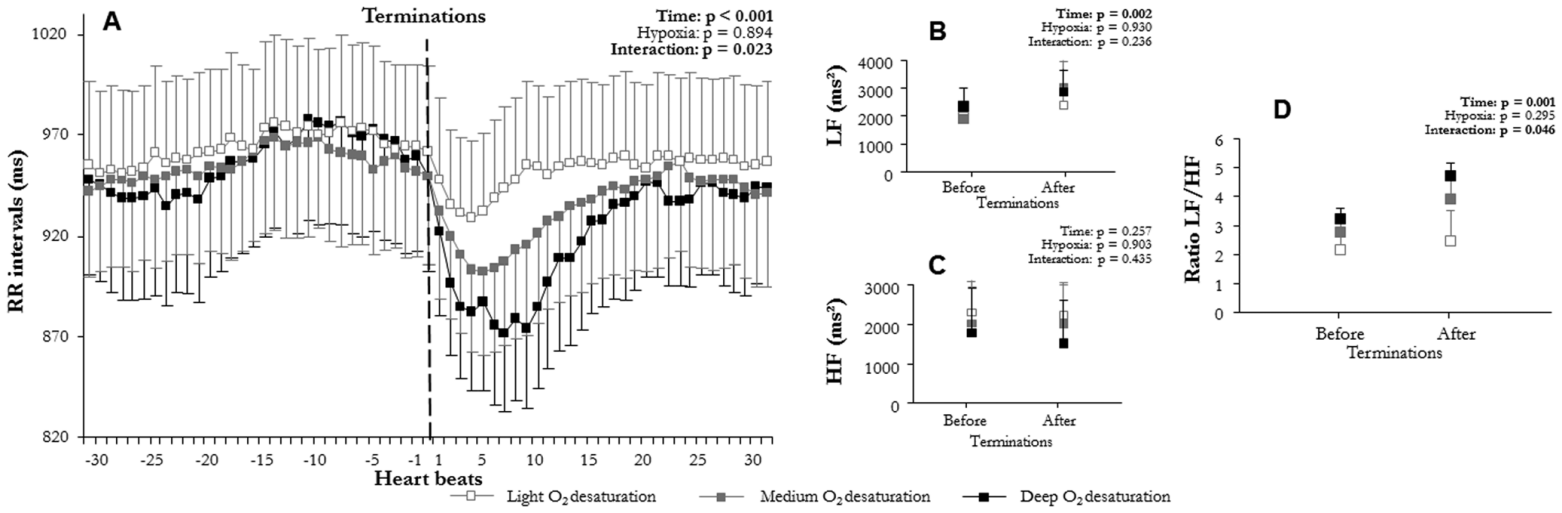
Comparison of autonomic modulation at apnea/hypopnea terminations according to oxygen desaturation: (A) RR intervals (RR), (B) low (LF^WV^) and (C) high frequency (HF^WV^) wavelet power, and (D) LF^WV^/HF^WV^ ratio before and after apnea/hypopnea terminations according to light oxygen desaturation (min SaO_2_≥90%) medium (90>min SaO_2_>85%) and deep oxygen desaturation (min SaO_2_≤85%) (mean ± SEM). An overall effect on RR intervals, LF^WV^, and LF^WV^/HF^WV^ ratio appeared between before and after apnea/hypopnea terminations. Apneas/hypopneas induced a sympathetic-dependent RR decrease, which was higher when apneas/hypopneas produced deep oxygen desaturation.

**Figure 7:**
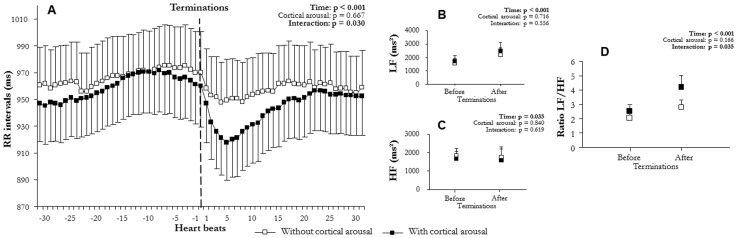
Comparison of autonomic modulation at apnea/hypopnea terminations according to cortical arousals: (A) RR intervals (RR), (B) low (LF^WV^) and (C) high frequency (HF^WV^) wavelet power, and (D) LF^WV^/HF^WV^ ratio before and after apnea/hypopnea terminations during no cortical and cortical arousals (mean ± SEM). An overall effect on RR intervals, LF^WV^, and LF^WV^/HF^WV^ ratio appeared between before and after apnea/hypopnea terminations. Apneas/hypopneas induced a sympathetic-dependent RR decrease, which was higher when apneas/hypopneas produced cortical arousals.

Mean RR varied significantly with time (F(2,6) = 58.3, p<0.001, Figure 2A; F(1,15) = 58.2, p<0.001, Figure 3A; F(2,12) = 47.6, p<0.001, Figure 4A; F(2,10) = 47.7, p<0.001, Figure 5A; F(2,9) = 73.6, p<0.001, Figure 6A; F(1,15) = 52.5, p<0.001, Figure 7A). Mean RR did not vary with sleep stage (F(2,6) = 0.1, p = 0.952, interaction: F(2,6) = 0.1, p = 0.804), type (F(1,15) = 0.1, p = 0.913, interaction: F(1,15) = 0.6, p = 0.429), or apnea/hypopnea duration (hypopneas: F(2,12) = 0.1, p = 0.924, interaction: F(2,12) = 1.0, p = 0.381; apneas: F(2,12) = 0.1, p = 0.923, interaction: F(2,12) = 1.0, p = 0.385). Mean RR was also unchanged by hypoxia (F(2,9) = 0.1, p = 0.894) and CA (F(1,15) = 0.1, p = 0.667), but an interaction of these effects was observed (hypoxia: F(2,9) = 4.4, p = 0.023 and CA presence: F(1,15) = 5.3, p = 0.030). Thus, mean RR significantly decreased at apnea/hypopnea termination from its level during apneas/hypopneas, and the decrease was greater with CA than with no CA. This RR decrease was also greater with deep hypoxia than with no hypoxia. However, sleep stage, type, and duration of respiratory events did not modulate RR decrease.

LF^WV^ varied significantly with time (F(2,6) = 31.4, p<0.001, Figure 2B; F(1,15) = 32.4, p<0.001, Figure 3B; F(2,12) = 12.4, p<0.001, Figure 4B; F(2,10) = 12.3, p = 0.001, Figure 5B; F(2,9) = 12.3, p<0.001, Figure 6B; F(1,15) = 26.4, p<0.001, Figure 7B). None of the tested effects or their interactions were significant (sleep stage: F(2,6) = 0.1, p = 0.864, interaction: F(2,6) = 0.1, p = 0.921; type: F(1,15) = 0.4, p = 0.515, interaction: F(1,15) = 0.3, p = 0.582; duration: hypopneas: F(2,12) = 0.2, p = 0.826, interaction: F(2,12) = 1.9, p = 0.189; apneas: F(2,10) = 0.2, p = 0.836, interaction: F(2,10) = 1.8, p = 0.189); hypoxia: F(2,9) = 0.1, p = 0.236, interaction: F(2,9) = 1.5, p = 0.236; CA: F(1,15) = 0.1, p = 0.716, interaction: F(1.15) = 0.3, p = 0.556). LF^WV^ increased at apnea/hypopnea termination, but the LF^WV^ increase did not differ according to sleep stage, type and duration, hypoxia, or CA presence.

HF^WV^ varied significantly with time only in the comparisons with apnea/hypopnea type and CA presence (F(1,15) = 16.0, p = 0.001, Figure 3C and F(1,15) = 26.4, p = 0.035, Figure 7C respectively), and not in other comparisons (F(2,6) = 0.1, p = 0.489, Figure 2C; F(2,12) = 1.3, p = 0.268, Figure 4C; F(2,10) = 0.1, p = 0.983, Figure 5C; F(2,9) = 12.3, p = 0.002, Figure 6C). None of the effects tested or their interactions were significant (sleep stage: F(2,6) = 0.5, p = 0.972, interaction: F(2,6) = 0.4, p = 0.651; type: F(1,15) = 0.1, p = 0.879, interaction: F(1,15) = 0.2, p = 0.653; duration: hypopneas: F(2,12) = 0.1, p = 0.983, interaction: F(2,12) = 1.7, p = 0.200; apneas: F(2,10) = 1.3, p = 0.267, interaction: F(2,10) = 1.7, p = 0.200); hypoxia: F(2,9) = 0.1, p = 0.930, interaction: F(2,9) = 1.5, p = 0.236; CA: F(1,15) = 0.1, p = 0.840, interaction: F(1.15) = 0.3, p = 0.619).

LF^WV^/HF^WV^ ratio varied significantly with time (F(2,6) = 14.8, p = 0.001, Figure 2D; F(1,15) = 19.1, p = 0.001, Figure 3D; F(2,12) = 7.9, p = 0.009, Figure 4D; F(2,10) = 7.7, p = 0.010, Figure 5D; F(2,9) = 16.7, p = 0.001, Figure 6D; F(1,15) = 26.8, p = 0.001, Figure 7D). LF^WV^/HF^WV^ ratio did not vary significantly with sleep stage (F(2,6) = 0.3, p = 0.780), type (F(1,15) = 0.2, p = 0.644), or duration (hypopneas: F(1,12) = 0.1, p = 0.961 and apneas: F(1,10) = 0.1, p = 0.931), and no interaction was observed (F(2,6) = 0.1, p = 0.936; F(1,15) = 1.0, p = 0.322; F(2,12) = 0.2, p = 0.814, Figure 4D; F(2,10) = 0.2, p = 0.790 respectively). LF^WV^/HF^WV^ ratio also did not vary with hypoxia (F(2,9) = 1.3, p = 0.295, Figure 6D) and CA (F(1,15) = 4.59, p = 0.035, Figure 7D), but showed an interaction effect (F(2,9) = 4.6, p = 0.035 and F(2,15) = 4.6, p = 0.035, respectively). Thus, LF^WV^/HF^WV^ ratio increased at apnea/hypopnea termination over the level during apneas/hypopneas. The only differences in the increase in LF^WV^/HF^WV^ ratio were according to the presence of CA and deep oxygen desaturation compared to their absence.

### Autonomic activity during apnea/hypopnea and its relationship with cortical arousal occurrence

To assess whether autonomic activity during apneas/hypopneas was associated with CA occurrence, multivariate logistic regression analysis was applied to investigate the relationship between the autonomic index of HRV and subsequent CA. Results showed a significant relationship between CA occurrence and LF^WV^ (χ^2^ = 7.42, *p* = 0.006) and LF^WV^/HF^WV^ ratio (χ^2^ = 4.80, *p* = 0.029), independently of mini and mean SaO_2_, type and duration, interval inter-events, sleep stage, and subject. No significant results were noted for RR mean (χ^2^ = 1.35, *p* = 0.244) or HF^WV^ (χ^2^ = 0.01, *p* = 0.906).

Using tertiles of HRV data, the independent relationships between CA occurrence, LF^WV^, and LF^WV^/HF^WV^ ratio remained significant (Table 2). For LF^WV^, the independent risk of CA occurrence increased from medium (OR = 1.34 (1.06–1.71), *p* = 0.016) to high tertile (OR = 1.92 (1.45–2.57), *p*<0.001). For LF^WV^/HF^WV^ ratio, the independent risk increased from medium (OR = 1.55 (1.21–1.98), p<0.001) to high tertile (OR = 1.92 (1.46–2.52), *p*<0.001). No significant relationship was found between CA occurrence, HF^WV^, and RR mean during apneas/hypopneas.

**Table 2:** Odds ratios (OR) and 95% confidence intervals (95% CI) for the occurrence risk of subsequent cortical arousal at apnea/hypopnea terminations.

Characteristics	Crude OR (95% CI)	*p*	Adjusted OR (95% CI)	*p*
RR mean 1st tertile	1	–	1	–
2nd tertile	1.12 (0.90–0.37)	**0.310**	0.86 (0.64–1.16)	**0.332**
3rd tertile	1.51 (1.22–1.90)	**0.002**	0.92 (0.58–1.44)	**0.703**
LF^WV^ 1st tertile	1	–	1	–
2nd tertile	1.32 (1.07–1.63)	**<0.001**	1.34 (1.06–1.71)	**0.016**
3rd tertile	1.79 (1.44–2.23)	**<0.001**	1.92 (1.45–2.57)	**<0.001**
HF^WV^ 1st tertile	1	–	1	–
2nd tertile	0.75 (0.61–0.93)	**0.003**	0.81 (0.62–1.07)	**0.143**
3rd tertile	1.04 (0.83–1.29)	**0.766**	0.98 (0.69–1.39)	**0.892**
LF^WV^/HF^WV^ ratio 1st tertile	1	–	1	–
2nd tertile	1.45 (1.17–2.25)	**<0.001**	1.55 (1.21–1.98)	**<0.001**
3rd tertile	1.80 (1.45–2.25)	**<0.001**	1.92 (1.46–2.52)	**<0.001**

Logistic multivariate regressions were performed between each autonomic cardiac index during apneas/hypopneas (expressed in tertiles) and CA occurrence, with adjustment for the potential effects on CA occurrence of minimum and average oxygen saturation, apnea/hypopnea type, sleep stage, interval inter-events, and subjects.

## Discussion

First, the results of this study showed that sympathetic modulation at apnea/hypopnea termination is modulated by the arousal process and hypoxia rather than by sleep stage or apnea/hypopnea type and duration in obstructive sleep apnea/hypopnea syndrome (OSAHS) patients. Greater sympathetic activation in response to apneas/hypopneas is concomitant with the presence of cortical arousal (CA) or deep hypoxia. Second, when cardiac sympathetic indexes of heart rate variability (HRV) were high during apneas/hypopneas, the risk of CA occurrence was magnified (or twice). This indicates that cardiac sympathetic dominance during apneas/hypopneas is related to facilitated CA occurrence. However, the exact relationship between sympathetic activity during apneas/hypopneas and subsequent CA remains unknown.

### Sleep autonomic modulation at apnea/hypopnea termination

Spicuzza and collaborators [9] used time frequency analysis to detect oscillations in parasympathetic and sympathetic cardiac activity during apneas, corroborating other studies [7], [8]. These oscillations are characterized mainly by parasympathetic dominance during apneas and sympathetic modulation at apneas termination. Using a similar time frequency method, the present study confirmed that sympathetic modulation dominates at apnea/hypopnea termination (increase in both LF^WV^ and LF^WV^/HF^WV^ ratio), with a small decrease in parasympathetic activity (HF^WV^). This parasympathetic withdrawal appeared significant only in comparison with the higher number of subjects, e.g. comparison with and without CA (n = 16), and according to apnea/hypopnea type (n = 16). In addition, using Fourier transform, a less sensitive method to study transit changes in RR, previous studies [8], [9] showed no change in spectral HF power. This parasympathetic withdrawal at apnea/hypopnea termination appeared to be small and difficult to detect. Moreover, OSAHS patients presented a predominant cardiovascular sympathetic activity with decreased parasympathetic control [4], [5]. This could explain the difficulty in detecting transit changes in the parasympathetic arm in this OSAHS population, even though we used time frequency analysis. Thus, apnea/hypopnea termination induces a cardiac reflex activation, implying essentially sympathetic drive whereas parasympathetic withdrawal is less marked, parasympathetic activity been more related to physiological changes during apneas/hypopneas [10], [22].

### Control of sympathetic modulation at apnea/hypopnea termination

Autonomic dysfunction is recognized to contribute to cardiovascular consequences in these OSAHS patients [3]. To better understand the relationship between apneas/hypopneas and cardiac autonomic dysfunction in OSAHS population, we specifically examined apnea/hypopnea termination to explore the mechanisms that control sympathetic cardiac modulation by HRV analysis. We did not exclude the effect of parasympathetic dysfunction in OSAHS patients on cardiovascular morbidity and mortality [3]. However, modulation of the parasympathetic arm during apneas/hypopneas has been well studied [10], [22], whereas the factors that modulate sympathetic cardiac modulation at apnea/hypopnea termination have been less studied, with inconsistent results [8]–[10]. Moreover, we have previously shown that sympathetic sleep fragmentation was associated with elevated nocturnal and diurnal systolic blood pressure and higher risk of systolic hypertension [6].

Of the several factors tested in this study, sleep stage, apnea/hypopnea duration, and respiratory event type showed no effect on sympathetic cardiac modulation: irrespective of sleep stage, type, and duration, apneas/hypopneas produced the same level of cardiac sympathetic modulation (Figure 2–4).

The absence of sleep stage difference concurs with previous studies, which showed that exteroceptive stimulations [11] or periodic legs movements [12] produce the same level of sympathetic cardiac activation regardless of sleep stage. Regarding apneas/hypopneas, sympathetic cardiac change was described as higher [10] or lower [8], [9] in paradoxical sleep than in other sleep stages. These discrepancies could be explained by different methodological approaches (Fourier [9] vs. wavelet transform in our study), or by the previous small number of apnea/hypopnea observations (61 events [10] vs. 1524 in 7 subjects in our study). Thus, apneas/hypopneas may produce the same level of sympathetic cardiac modulation regardless of sleep stage (Figure 2).

Hierarchy in the arousal process and its close relationship with sympathetic cardiac modulation has been previously reported in several conditions of sleep fragmentation, including spontaneous arousal [12], periodic leg movements [23], and auditive [24] or nociceptive stimulation [10]. However, it remains unclear whether this relationship holds true for OSAHS patients presenting recurrent apneas/hypopneas during sleep, basal autonomic dysfunction [4], or abnormal autonomic responses to stress [25]. Moreover, previous studies showed inconsistent results: CA had an effect on sympathetic cardiac modulation [9] or not [8], [10]. However, studies with negative results used a low number of apneas/hypopneas (only 61 events [10] vs. 2972 in 7 patients in the present study) or Fourier transform, known to be less effective than the time frequency method to study transient variation in RR [8]. Our results clearly showed that sympathetic cardiac modulation varied contingently with cortical reactivity. Cardiac reactivity was higher when apneas/hypopneas produced CA, although preserved in the absence of subsequent CA. Thus, the arousal process appeared to be a major modulator of sympathetic modulation to apneas/hypopneas.

Then, we hypothesized that severity of respiratory events could be expressed as length or type of respiratory events, but our result rather showed that both type and duration of respiratory events do no bring information on cardiac sympathetic modulation. Our results confirm previous data [8] showing no direct effect of respiratory event type, i.e. apneas vs. hypopneas, on sympathetic cardiac modulation (Figure 3–4). Concerning the duration of respiratory event, although we hypothesized that longer duration would increase cardiac sympathetic modulation, this was not the case.

Finally, recurrent nocturnal hypoxia, which is suspected to be a major factor of cardiac disease in OSAHS patients [3], [26], appears to have a strong influence on cardiac sympathetic modulation in response to apneas/hypopneas in our study. Indeed a min SaO_2_ decrease lesser than 90% was able to induce a significant cardiac sympathetic modulation increase (Figure 6). These results concur with those of a previous study showing a correlation between concomitant min SaO_2_ and sympathetic cardiac modulation in response to apneas/hypopneas [10]. However, several authors [27], [28] found no difference between hypoxia (mean SaO_2_: 88.8±0.4%) and normoxia (mean SaO_2_: 97.9±0.2%) in cardiac response to auditive stimulations during sleep in healthy volunteers. This discrepancy between their and our results could be explained by the elevated chemoreflex sensitivity in OSAHS patients, which causes cardiac sympathetic modulation in response to decrease in SaO_2_ [29]. In turn, this exposure to recurrent hypoxia and cardiac sympathetic modulation during sleep may also contribute to chemoreflex impairment in OSAHS patients [30]. Thus, elevated cardiac sympathetic activation in response to apneas/hypopneas is concomitant with strong chemoreflex activation, potentially leading to the increase in diurnal chemoreflex sensitivity in OSAHS population [30], [31].

This cardiac sympathetic modulation in response to respiratory events influenced by the arousal and hypoxia processes could represent a pathway contributing to cardiovascular disease risk in OSAHS. Recurrent hypoxia, known to be a strong factor of cardiac disease in OSAHS patients, can also act by other pathways, such as oxygen free radical production, inflammatory pathways, and impairment in the vascular endothelial function independently of activation of the sympathetic arm [3]. EEG sleep fragmentation, proposed as a potential contributor to hypertension, was related to an approximately 3 mmHg rise in diurnal systolic blood pressure in a population without sleep-disordered breathing [32] and recently, sleep fragmentation based on autonomic markers (pulse transit time) have been shown to be related to diurnal blood pressure increase and hypertension risk [33]. Our study supports the idea that the recurrent arousal and hypoxia processes, by cardiac sympathetic modulation in response to apneas/hypopneas, could contribute to a decrease in HRV [4], [33] and a predominant cardiovascular sympathetic activity that persists during wakefulness [5], increasing the cardiovascular disease risk in OSAHS populations.

### Relationship between sympathetic cardiac activity and subsequent cortical arousal

In the last part of this study, we examined the relationship between autonomic indexes of HRV during apneas/hypopneas and subsequent CA. Results showed that the spectral LF^WV^ power and LF^WV^/HF^WV^ ratio during apneas/hypopneas, sympathetic criteria of HRV, are related to CA occurrence at apnea/hypopnea termination, indicating that cardiac sympathetic dominance during apneas/hypopneas is related to a facilitated CA occurrence.

These results are in accord with studies that proposed sympathetic cardiac activity during sleep as a contributing factor to the CA occurrence process, suggested by observations obtained in both animals [13], [14] and humans [15], [16]. Other studies showed that spontaneous CA [17] and periodic leg movements [34] or bruxism events [35] are preceded by a rise in heart rate. Moreover, during apneas/hypopneas, although RR increase and parasympathetic activity dominates, Bonsignore [3], [17] reported two typical patterns: RR continued to increase during the apneas, and RR decreased before apnea termination. Although our study indicates a relationship between sympathetic dominance during apneas/hypopneas during and subsequent to CA, we did not identify the mechanism by which autonomic stimulation contributes to arousal or the stimuli at the origin of this sympathetic disequilibrium.

It therefore appears likely that variations in peripheral afferents information may play an important role in the acute autonomic response as part of a multifactorial process [36], such as hypoxemia [22] and hypercapnia [37], thoracic pressure swings [17], baroreflex control [16], or pulmonary afferent stimuli arising from the chest wall or the lung in response to intrathoracic pressure changes or from the upper airway in response to airflow obstruction.

### Limitations

Several limitations of this study should be considered. The method is limited for study of sympathetic activity: the LF^W^ is related to both sympathetic and parasympathetic activity. It is only by studying the relative changes in LF^W^ and HF^W^, and normalized indexes as LF^W^/HF^W^ ratio that an indication of sympathetic activity is obtained. Several studies have showed that LF/HF ratio and LF% allow approaching relative sympathetic activity, in response to tilt test under atropine [38] as well as to experimental pain [11], auditory stimulations [24] and bruxism episodes [35] during sleep. These studies are consistent with our interpretation of the present results. Second, we compared autonomic changes at terminations from autonomic level during apneas/hypopneas. This can be adapted to compare with stable baselines. Although stable baselines can be easily obtained in wakefulness or stable sleep periods in healthy subjects, but is more problematic in OSAHS patients, as most of these subjects go from one episode to the next one without real stable baseline. Third, we cannot exclude that CO_2_ retention, a factor that was not considered here, could contribute to these autonomic changes. However, a previous study showed that CO_2_ level did not affect cardiac modulation in response to spontaneous arousals in healthy volunteers [27]. At last, number of apnea/hypopnea observations during SWS, statistical analysis was performed on a small number subjects (7 subjects for comparison between sleep stages), because its remains difficult to obtain hypopnea/apneas during SWS, due to the small numbers. These issues need to be addressed in wide OSAHS populations in future studies.

## Conclusions

Apneas/hypopneas induce a cardiac sympathetic modulation influenced by the arousal and hypoxia processes rather than by sleep stage, total or partial upper airway occlusion, or occlusion duration. Higher sympathetic activation in response to apneas/hypopneas is concomitant with the presence of cortical arousal or a deep hypoxia compared to no cortical arousal or no hypoxia. Furthermore, cardiac sympathetic dominance during apneas/hypopneas is related to facilitated CA occurrence. Both the recurrent arousal and hypoxia processes by cardiac sympathetic modulation in response to apneas/hypopneas, could contribute to predominant cardiovascular sympathetic activity that persists during wakefulness, increasing the cardiovascular disease risk in OSAHS populations. On the other hand, sympathetic overactivity also may play an important role in the acute central response to apneas/hypopneas, and so in the sleep fragmentation.
